# Author Correction: Human eye-inspired soft optoelectronic device using high-density MoS_2_-graphene curved image sensor array

**DOI:** 10.1038/s41467-022-32730-1

**Published:** 2022-09-02

**Authors:** Changsoon Choi, Moon Kee Choi, Siyi Liu, Minsung Kim, Ok Kyu Park, Changkyun Im, Jaemin Kim, Xiaoliang Qin, Gil Ju Lee, Kyoung Won Cho, Myungbin Kim, Eehyung Joh, Jongha Lee, Donghee Son, Seung-Hae Kwon, Noo Li Jeon, Young Min Song, Nanshu Lu, Dae-Hyeong Kim

**Affiliations:** 1 Center for Nanoparticle Research, Institute for Basic Science (IBS), Seoul, 08826 Republic of Korea; 2 School of Chemical and Biological Engineering, Institute of Chemical Processes, Seoul National University, Seoul, 08826 Republic of Korea; 3 Center for Mechanics of Solids, Structures, and Materials, Department of Aerospace Engineering and Engineering Mechanics, University of Texas at Austin, 210 E 24th St, Austin, TX 78712 USA; 4 School of Mechanical and Aerospace Engineering, Seoul National University, Seoul, 08826 Republic of Korea; 5 Onfea Computing LLC, 204 Jackson Street, Newton, MA 02459 USA; 6 School of Electrical Engineering and Computer Science, Gwangju Institute of Science and Technology, Gwangju, 61005 Republic of Korea; 7 Division of Bio-imaging, Korea Basic Science Institute, Chun-Cheon, 24341 Republic of Korea; 8 Department of Electrical and Computer Engineering, Department of Biomedical Engineering, Texas Materials Institute, the University of Texas at Austin, Austin, TX 78712 USA

Correction to: *Nature Communications* 10.1038/s41467-017-01824-6, published online 21 November 2017

Since the publication of this work, Minsung Kim has changed their name from Min Sung Kim. This has now been amended.

