# Supplementary material for: Human eye-inspired soft optoelectronic device using high-density MoS2-graphene curved image sensor array
Source: Nat Commun. 2017 Nov 21;8:1664. doi: 10.1038/s41467-017-01824-6 (PMC5698290; doi:10.1038/s41467-017-01824-6)
Supplement: Supplementary file 1 — Supplementary Information [file 41467_2017_1824_MOESM1_ESM.pdf]

Supplementary Note 1. Optic simulation for various optical systems

The ray tracing simulations for each optical system were performed using commercial software (Zemax, USA). The spherical aberration induces mismatch between the curved focal plane and the flat image sensor (Supplementary Fig. 1a top), which requires a complicated lens configuration in current camera modules. For example, a Double Gauss lens consists of seven lenses for focusing the image on the planar image sensor, and it needs ~93.2 mm of lateral dimension (Supplementary Fig. 1a bottom). Detailed lens parameters such as radii, thicknesses, and materials are included in Supplementary Table 1. In addition, achieving accurate alignment between the individual lenses in the Double Gauss lens increases the fabrication cost. On the other hand, the CurvIS array using a single plano-convex lens (Supplementary Table 2) satisfies requirements to achieve the focus on the entire curved focal plane (Supplementary Fig. 1b). The optic design for this configuration is simple and compact (~29.3 mm) compared to the multi-lens configurations required for flat image sensors. This also dramatically reduces the production cost. In these simulations, F-number of 5 was selected as an aperture stop size. The blue, green, and red lines indicate the rays of light whose field-of-view are 0°, 14°, and 28°, respectively.

Imaging simulations for each optical system were also performed using the commercial software. The single plano-convex lens successfully focuses the image on the CurvIS array without distortion (Supplementary Fig. 2d) similar to the conventional Double Gauss lens (Supplementary Fig. 2c). On the other hand, an image focused on a flat image sensor array through a single plano-convex lens is blurred (Supplementary Fig. 2b).

Supplementary Note 2. Theoretical analysis of the soft optoelectronic device based on mechanics

Theoretical analysis is based on mechanics of membranes conformed to spherical surface. Since the Young's modulus of polyimide (PI) is in the GPa range and the device is ultrathin (51 nm; Fig. 1c) in comparison with the encapsulating PI layer (840 nm), the strain transfer between the PI encapsulation and the device is close to 100%[1]. As a result, we can simply regard the CurvIS array as a uniform and continuous PI film in this theoretical analysis. For a uniform and continuous circular thin film of outer radius R_f that fully adheres to a rigid sphere of radius R_d, the linear elasticity solution of the strain distribution in the film is given by[2]

$$\varepsilon_{rr} = \left(\frac{R_f}{R_d}\right)^2 \frac{(1-\nu)-(1-3\nu)\left(r/R_f\right)^2}{16} + \frac{z}{R_d},$$
$$\varepsilon_{\theta\theta} = \left(\frac{R_f}{R_d}\right)^2 \frac{(1-\nu)-(3-\nu)\left(r/R_f\right)^2}{16} + \frac{z}{R_d}, \tag{1}$$

where r is the radial coordinate, z is the thickness coordinate with the middle plane of the film being the origin, and ν is the Poisson's ratio of the film. In our experiments, $\nu = 0.34$ is used for PI. The radius of the hemispherical dome is $R_d = 11.34$ mm, the radius of the truncated film is $R_{fs} = 3.5$ mm, the radius of the untruncated film is $R_{fl} = 9.3$ mm, and the total thickness of the device is $h = 891$ nm. Since $h/R_d \ll (1-\nu)\left(R_f/R_d\right)^2/16$, the strain dependence on thickness is negligible in our problem. Therefore, in the following discussion, we neglect the z/R_d terms in Supplementary Eq. (1).

The radial and hoop strain distributions for the truncated and untruncated films are plotted in Fig. 2b-e. R_f plays a significant role in determining the strains in the film. The untruncated film is subjected to higher radial and hoop strains, both tensile and compressive. The maximum radial strain in the film occurs at the edge ($r = R_f$), whereas the maximum hoop strain is found at the center ($r = 0$) of the film. Plugging the r's into Supplementary Eq.

(1), we find that both maximum strains have a quadratic relation with R_f/R_d.

$$\varepsilon_{\mathrm{rr,max}} = \left(\frac{R_f}{R_d}\right)^2 \frac{\nu}{8}, \qquad \varepsilon_{\theta\theta,\mathrm{max}} = \left(\frac{R_f}{R_d}\right)^2 \frac{(1-\nu)}{16} \tag{2}$$

The maximum radial and hoop strains of truncated and untruncated films are plotted as functions of R_f/R_d in Fig. 2f. Maximum compressive strains occur at the edge of the films. The much lower compressive strain in the truncated film can effectively prevent buckling and folding of the film (Supplementary Fig. 4).

Supplementary Note 3. Analytical solution of interfacial tractions between implantable devices and the artificial eye model.

For a film of radius R_f and thickness H attached to the eye model of radius R_d, the interfacial traction deforms the eye model, as evident in Fig. 4c. As we assume the eye model (*i.e.*, a bilayer hemispherical shell) to be rigid for a quick estimation of the interfacial traction, the estimated result would be an upper limit and would be more accurate for lower interfacial traction which induces smaller deformation in the eye model. For full conformability to the rigid eye model, the adhesion energy of the interface W_{ad} must satisfy[2]

$$W_{ad} \geq EH\left[\frac{1}{128}\left(\frac{R_f}{R_d}\right)^4 - \frac{H^2}{12(1-\nu)R_d^2}\right] \tag{3}$$

where E is the Young's modulus of the film. Assuming a rectangular traction separation relation, the interfacial traction σ can be estimated as

$$\sigma_c = \frac{W_{ad}}{\delta_c} \tag{4}$$

where the maximum separation is assumed to be $\delta_c = 20$ μm. In our experiments (Fig. 4c and

Supplementary Fig. 13), the radii of the soft optoelectronic device and the flexible film are R_f = 9.3 mm. The wafer-based electronics is square but we assume it is a circular film of radius R_f = 5 mm. The material properties, required adhesion energy and interfacial traction of the three cases are listed in Supplementary Table 3.

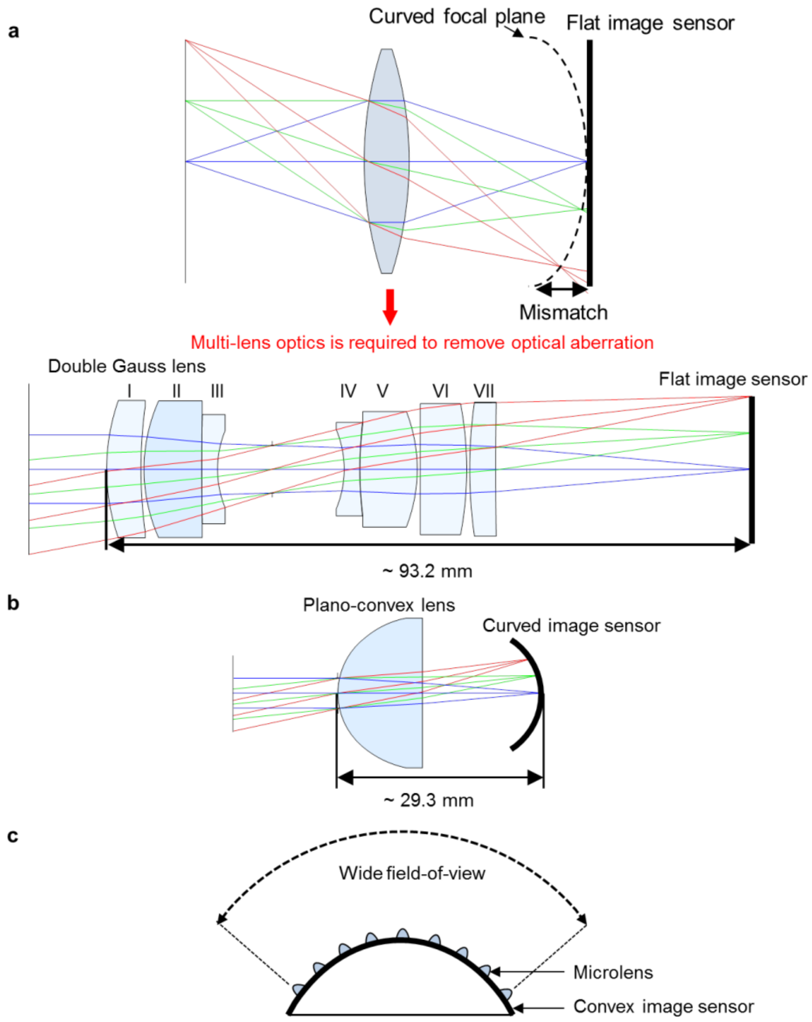

Supplementary Figure 1 | Ray tracing simulation of each optical system. (**a**) Optical aberration originated from the mismatch between the curved focal plane and the flat image sensor (top). Conventional Double Gauss lens to remove optical aberration for the flat image sensor (bottom). (**b**) Simplified optics consisting of a single plano-convex lens for the curved image sensor. (**c**) Wide field-of-view of the conventional hemispherically curved image sensor array[3].

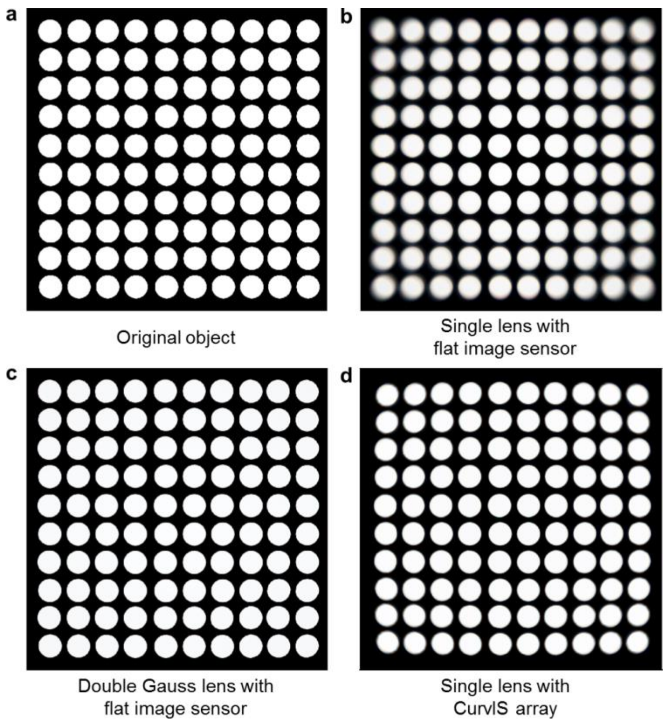

Supplementary Figure 2 | Imaging simulation. (**a**) Original object. (**b-d**) images focused by the three optical systems; single plano-convex lens with the flat image sensor array (**b**), Double Gauss lens with the flat image sensor array (**c**), and single plano-convex lens with the CurvIS array (**d**).

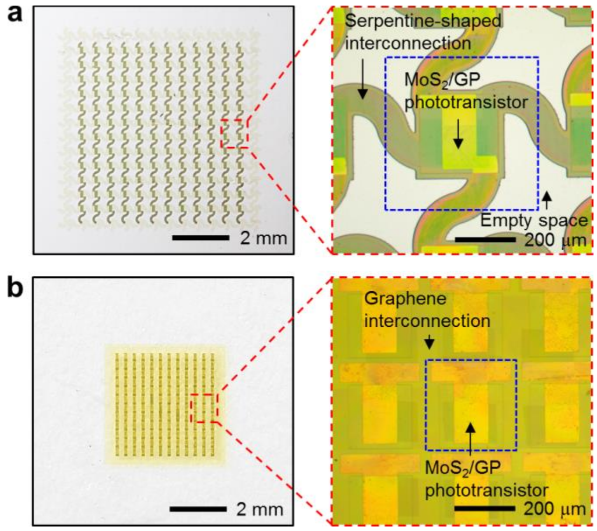

Supplementary Figure 3 | Device array design with and without serpentine-shaped interconnection. (**a**) Image of the device array with serpentine-shaped interconnections (left) and its magnified view (right). (**b**) Image of the compact device array without serpentine-shaped interconnections (left) and its magnified view (right). Blue dashed box indicates a single pixel.

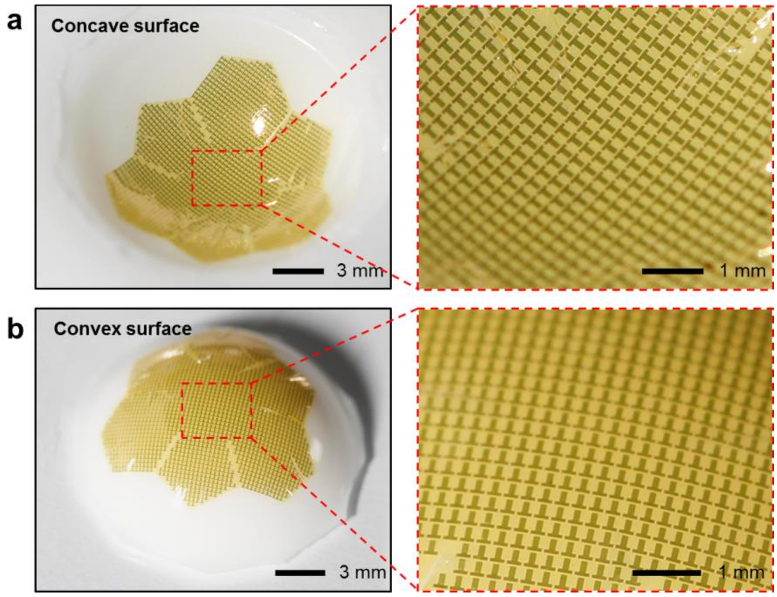

Supplementary Figure 4 | The curved image sensor array on the concave and convex hemisphere. (**a**) Optical camera image of the CurvIS array on a concave surface (left) and its magnified view (right). (**b**) Optical camera image of the CurvIS array on a convex surface (left) and its magnified view (right).

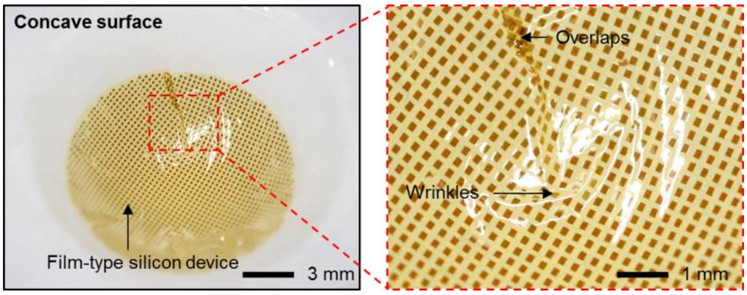

Supplementary Figure 5 | Circular film-type device array on the concave hemisphere. Optical camera image of the conventional film-type silicon (1.25 μm) photodiode array on a concave surface (left) and its magnified view (right).

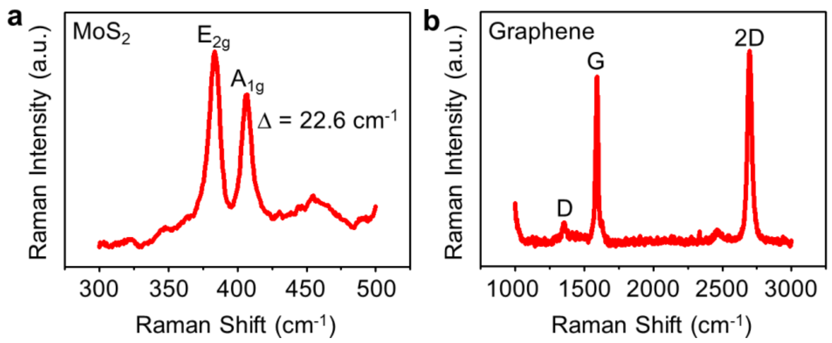

Supplementary Figure 6 | Characterization of synthesized MoS_2 and graphene film. (**a**) Raman spectrum of MoS_2 film. (**b**) Raman spectrum of graphene film.

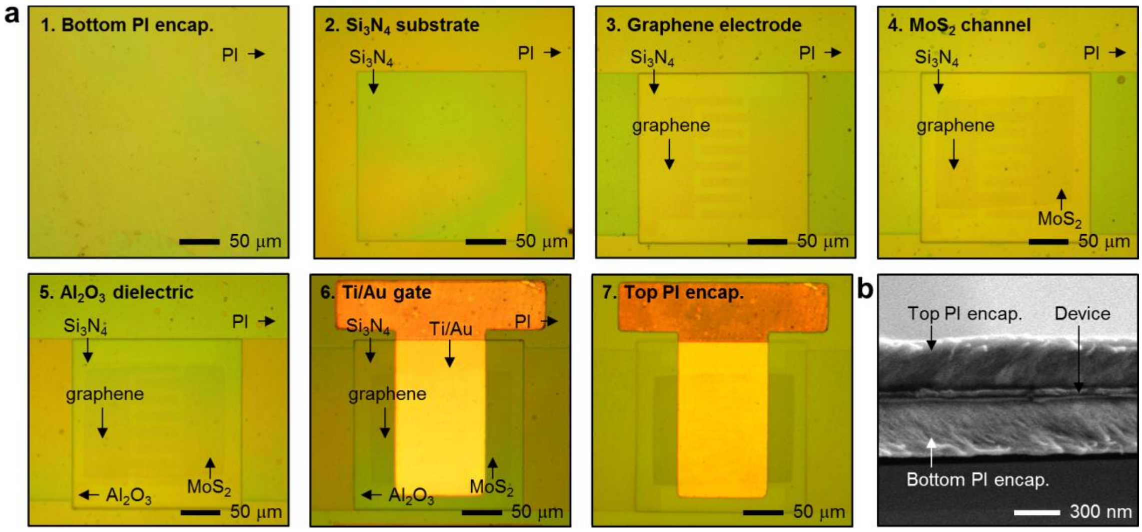

Supplementary Figure 7 | Fabrication of the phototransistor array based on the MoS_2-graphene heterostructure. (**a**) Optical microscope images for showing the fabrication process of a single phototransistor. (**b**) Scanning electron microscope image of the vertical structure of the device and the top and bottom PI encapsulations.

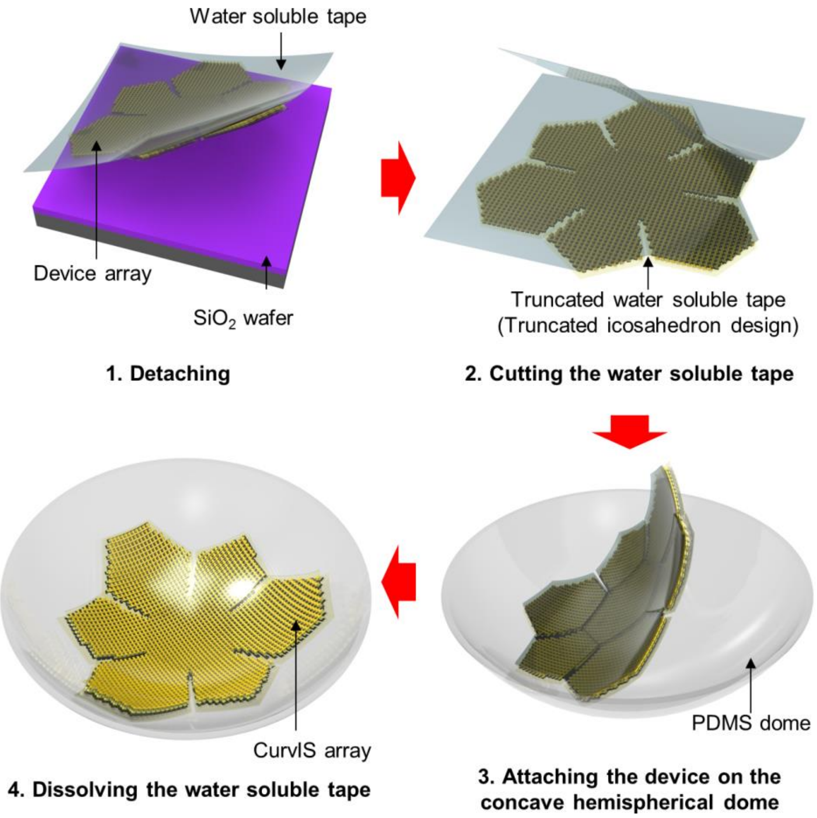

Supplementary Figure 8 | Integration of the curved image sensor array on the hemispherical surface. Method for transferring the MoS_2-graphene-based phototransistor array to the concave hemispherical dome.

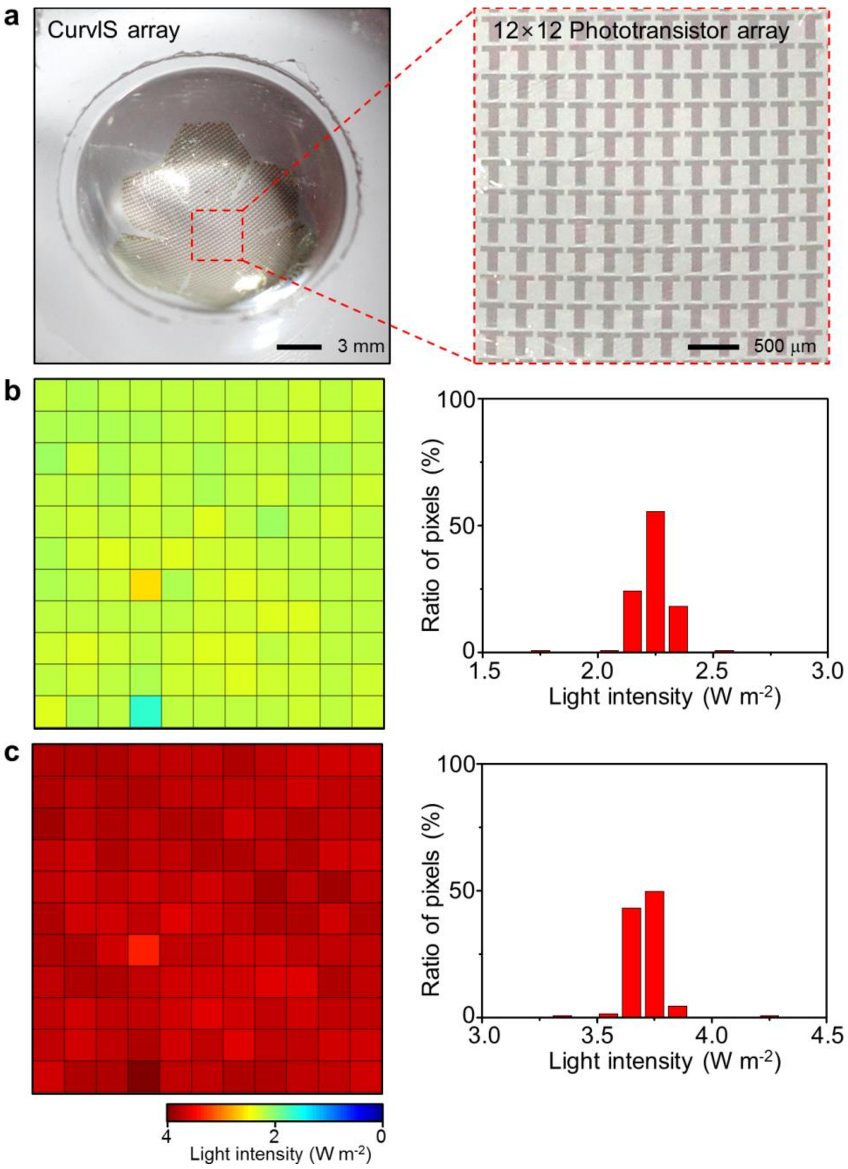

Supplementary Figure 9 | Characterization of the phototransistor array. (**a**) Optical camera image of the CurvIS array on the transparent concave hemispherical dome (left) and its magnified image showing the 12×12 phototransistor array (right). (**b,c**) Spatial distribution of measured light intensities using the calibrated phototransistor array (left) and its statistical analysis (right) under illumination of different light intensities (2.2 W m^{-2} and 3.7 W m^{-2} at 515 nm; for **b** and **c**, respectively).

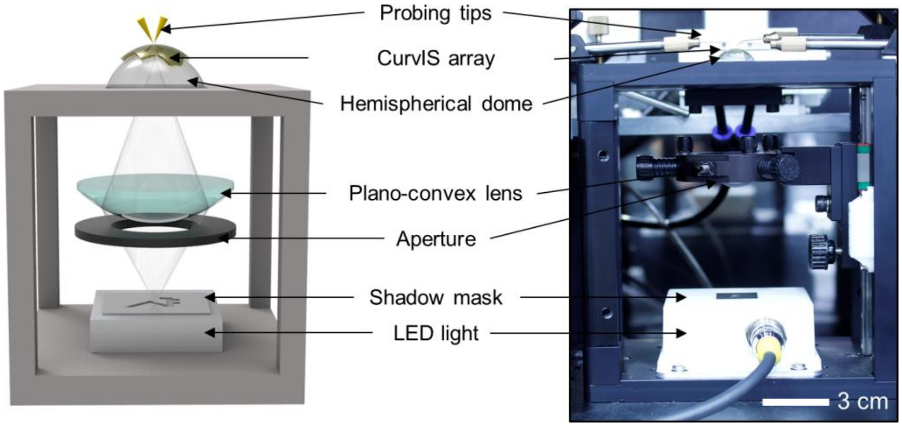

Supplementary Figure 10 | Experimental setup for imaging. Schematic illustration (left) and optical camera image (right) of the experimental setup. The setup consists of a white LED blocked with a metal shadow mask, an aperture, a plano-convex lens, the CurvIS array, and probing tips connected to a parameter analyzer.

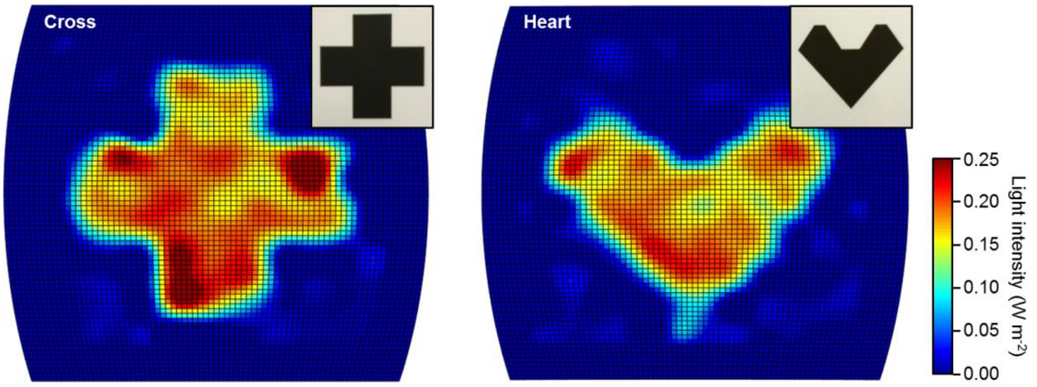

Supplementary Figure 11 | Imaging results using the curved image sensor array. Cross- and heart- shaped images obtained by the CurvIS array. Insets show original patterns.

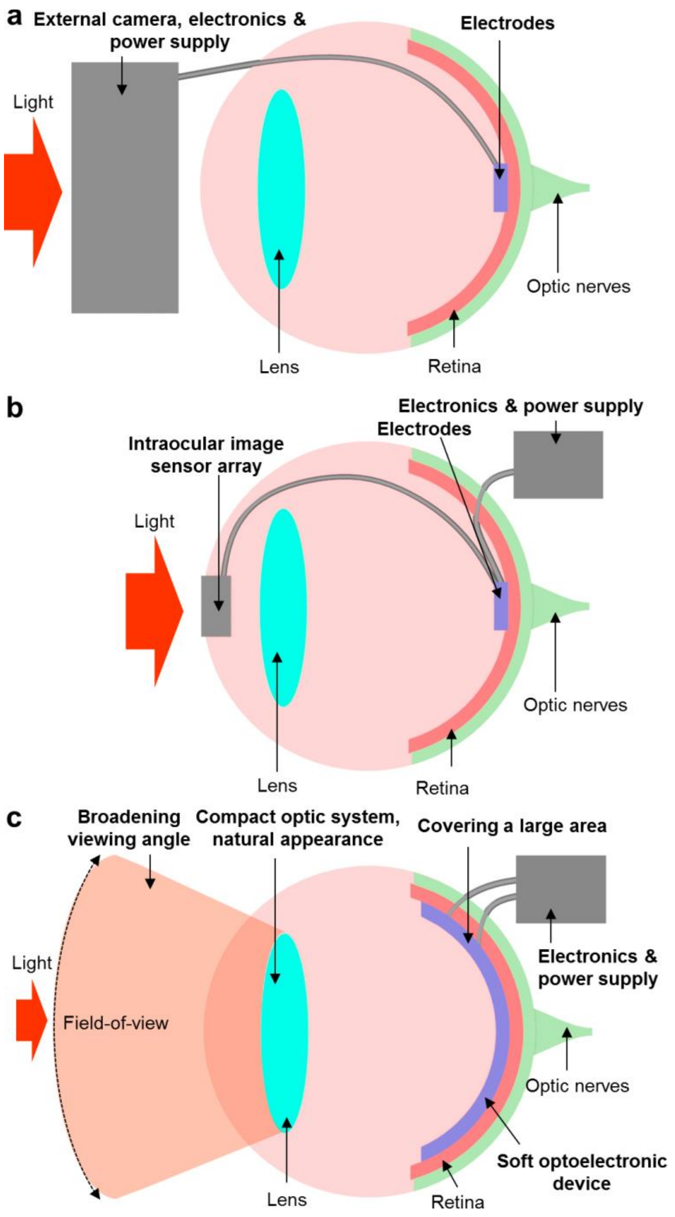

Supplementary Figure 12 | Schematic ocular structure implanted with retinal prostheses and the soft optoelectronic device. (**a-c**) Schematic drawing showing the ocular structure implanted with conventional retinal prostheses (*e.g.*, wearable head-mounted camera (**a**) and intraocular image sensor array (**b**)) and the soft optoelectronic device (**c**).

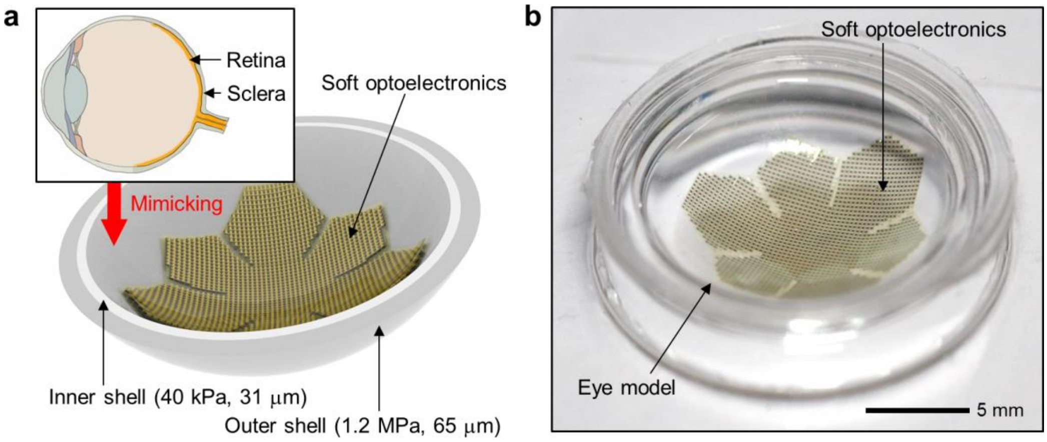

Supplementary Figure 13 | Eye model for analyzing the retinal deformation. (**a**) Schematic illustration of the double-layered eye model that mimics retina (20 kPa)[4] and sclera (1.84 MPa)[5] in human eye. (**b**) Optical camera image of the eye model attached with the soft optoelectronic device.

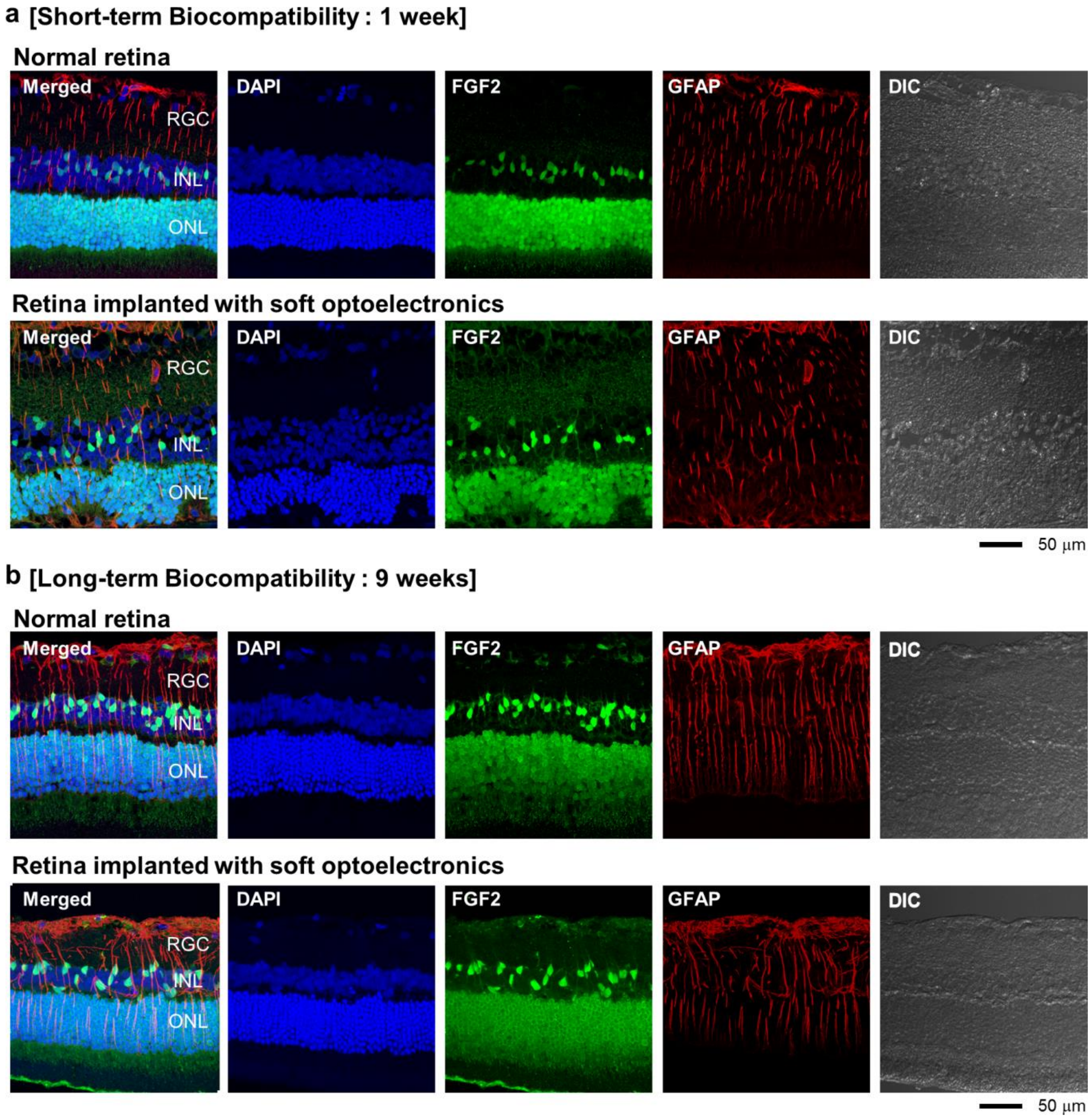

Supplementary Figure 14 | Biocompatibility of the soft optoelectronic device. (a,b) The histological staining data (DAPI, FGF2, and GFAP) and differential interference contrast (DIC) microscope image of the normal retina and the retina implanted with the soft optoelectronic device for the short-term (**a**) and long-term (**b**) period.

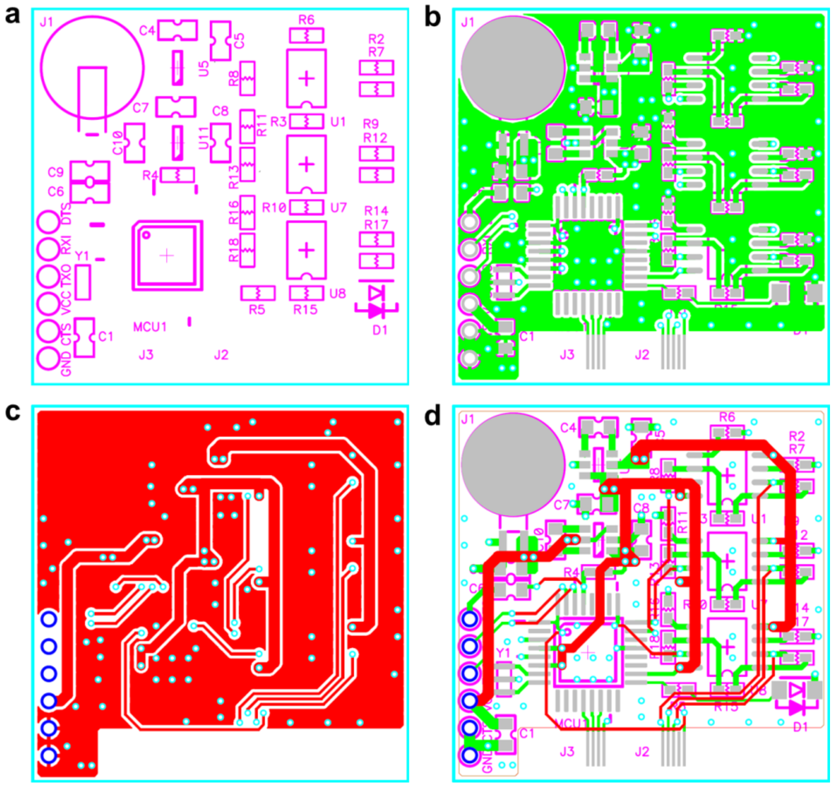

Supplementary Figure 15 | Soft flexible printed circuit board (soft FPCB). (a-d) Layout of the soft FPCB showing the comprising components (**a**), top connection map (**b**), bottom connection map (**c**), and merged connection map (**d**).

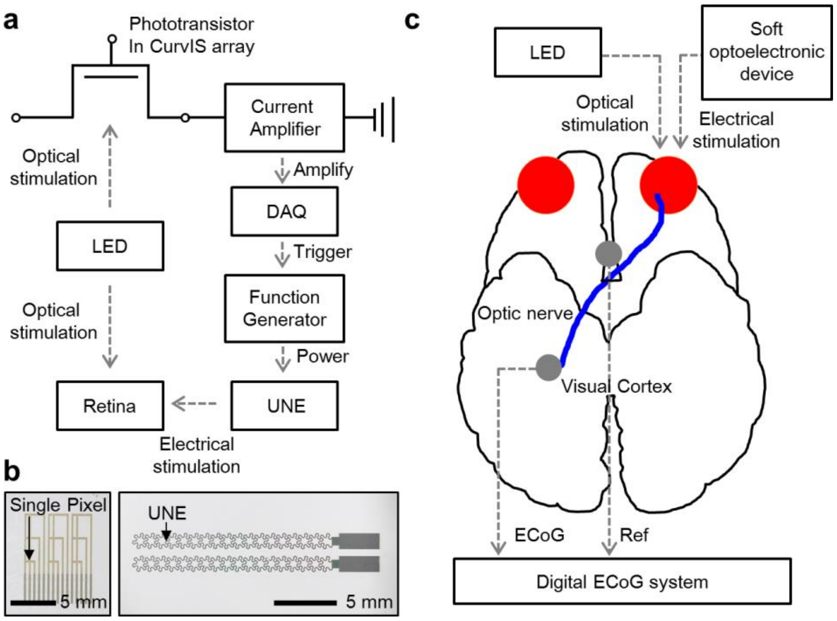

Supplementary Figure 16 | Experimental setup for the neural stimulation. (**a**) Block diagram that explains the experimental setup and sequence for detecting the external light and for stimulating the retina. (**b**) Optical camera image of the MoS_2-graphene-based phototransistor for detecting light (left) and the UNE for stimulating the retinal nerves (right), both of which are used for the *in vivo* animal experiment. (**c**) Schematic drawing of the experimental setup for stimulating the retinal nerves and for recording neural signals from the visual cortex.

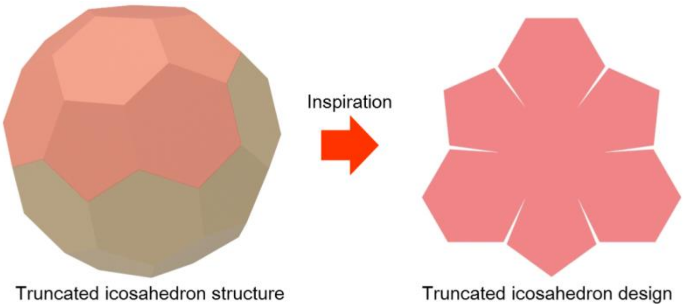

Supplementary Figure 17 | Truncated icosahedron design. The truncated icosahedron device array design inspired by the truncated icosahedron structure.

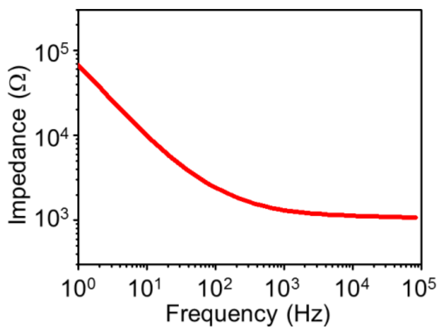

Supplementary Figure 18 | Characterization of the UNE. Impedance measurement of the UNE in the phosphate buffered solution.

lens.

Table 1 | Lens information of Double Gauss lens.					
**Surface**	**Label of lens**	**Radius (mm)**	**Thickness (mm)**	**Material**	**Semi-diameter (mm)**
Object		Infinity	Infinity	-	Infinity
1	I	34.333	4.435	N-BASF2	10.061
2		78.925	0.381	-	9.193
3	II	27.554	7.452	N-LAK8	10.000
4	III	592.999	2.032	SF2	8.000
5		16.807	7.036	-	5.625
Stop		Infinity	9.276	-	3.471
7	IV	-16.965	2.032	SF2	5.784
8	V	69.433	7.394	N-LAK33	6.843
9		-25.644	0.381	-	8.454
10	VI	Infinity	5.989	N-LAK33	8.888
11		-58.641	0.381	-	9.579
12	VII	79.263	3.348	N-LAK8	9.771
13		699.404	33.035	-	9.797
Image		Infinity	-	-	10.679

Supplementary Table 1 | Information of each lens component in the Double Gauss lens. The radii, thicknesses, materials, and semi-diameters of each lens used in the Double Gauss lens.

Table 2 | Lens information of measurement setup				
**Surface**	**Radius (mm)**	**Thickness (mm)**	**Material**	**Semi-diameter (mm)**
Object	Infinity	Infinity	-	Infinity
Stop	Infinity	0.000	-	2.532
1	13.127	12.220	N-BK7	12.700
2	Infinity	17.045	-	3.653
Image	-11.340	-	-	9.500

Supplementary Table 2 | Information of the plano-convex lens in the optical characterization setup. The radii, thicknesses, materials, and semi-diameters of lens used in the optical system for the CurvIS array.

Table 3 | Material properties, required adhesion energy, and interfacial traction						
	**Materials**	**Young's Modulus (GPa)**	**Radius (mm)**	**Thickness (μm)**	**W_{ad} (J m^{-2})**	**Interfacial traction (Mpa)**
Soft optoelectronics	PI	2.5	9.3	1.383	12.22	0.61
Flexible film	Al	69	9.3	15	3658	183
Wafer-based electronics	Si	165	5.0	525	429079	2270

Supplementary Table 3 | Information of analytical solution of interfacial traction. Required interfacial adhesion energy and tractions to fully conform three different types of implantable devices to the eye model.

Item	Model	Item	Model
MCU1	ATMEGA328P	Y1	8 MHz Resonator
J1	MS920SE-FL27E	D1	LY N971-HL-1
U1, U7, U8	LT1462	R2, R9, R14	ELE-R1608F, 5 MΩ
U5	ltc3200es6-5	R3, R8, R10, R13, R15, R18	ELE-R1608F, 1 MΩ
U11	ltc1983es6-5	R4	ELE-R1608F, 10 kΩ
C1, C4, C8, C9	ELE-C2012, 1 μF	R5	ELE-R1608F, 330 Ω
C5, C7, C10	ELE-C2012, 10 μF	R6, R11, R16	ELE-R1608F, 0.5 MΩ
C6	ELE-C2012, 0.1 μF	R7, R12, R17	ELE-R1608F, 10 MΩ

Supplementary Table 4 | Chip information of the electronic circuit. Detailed information of electronic components in the soft FPCB.

Supplementary References

1. Yang, S. & Lu, N. Gauge factor and stretchability of silicon-on-polymer strain gauges, *Sensors* **13**, 8577–8594 (2013).
2. Majidi, C. & Fearing, R. S. Adhesion of an elastic plate to a sphere. *Proc. R. Soc. A* **464**, 1309–1317 (2008).
3. Song, Y. M. *et al.* Digital cameras with designs inspired by the arthropod eye. *Nature* **497**, 95–99 (2013).
4. Jones, I. L., Warner, M. & Stevens, J. D. Mathematical modelling of the elastic properties of retina: a determination of Young's modulus. *Eye* **6**, 556–559 (1992).
5. Ko, M. W. L. Effect of corneal, scleral and lamina cribrosa elasticity, and intraocular pressure on optic nerve damages. *JSM Ophthalmol.* **3**, 1024 (2015).
