# Supplementary material for: Human eye-inspired soft optoelectronic device using high-density MoS2-graphene curved image sensor array
Source: Nat Commun. 2017 Nov 21;8:1664. doi: 10.1038/s41467-017-01824-6 (PMC5698290; doi:10.1038/s41467-017-01824-6)
Supplement: Supplementary file 3 — Description of Additional Supplementary Files [file 41467_2017_1824_MOESM3_ESM.pdf]

Description of Additional Supplementary Files

File Name: Supplementary Movie 1
Description: Soft eye model attached by the soft optoelectronic device. A soft optoelectronic device in a truncated icosahedron design conforms to a soft artificial eye model. The contour plot indicates the gap distance δ between the device and the eye model.

File Name: Supplementary Movie 2
Description: Soft eye model attached by the circular film device. A circular optoelectronic device with the same thickness and material as the device in Video 1 conforms to a soft artificial eye model. The contour plot indicates the gap distance δ between the device and the eye model.
